# Spinal Anesthesia for Cesarean Section in a Class III Obese Parturient: A Case Report

**DOI:** 10.7759/cureus.83214

**Published:** 2025-04-29

**Authors:** Yonggang Wang, Fan Lyu, Isadora C Chacon, Amit Patel, Natasa Grancaric, William Zhang

**Affiliations:** 1 Department of Anesthesiology, Metropolitan Hospital Center/New York Medical College, New York, USA; 2 Department of Anesthesiology, Beijing Chaoyang Hospital/Capital Medical University, Beijing, CHN; 3 Department of Anesthesiology, New York Medical College, New York, USA

**Keywords:** cesarean section, morbidly obese parturient, morbidly obese patients, parturient, spinal anesthesia

## Abstract

The rising prevalence of obesity, including among pregnant patients, presents challenges for anesthetic management during cesarean sections. We report the case of a parturient with class III obesity (BMI > 60) undergoing elective cesarean section successfully managed with single-shot spinal anesthesia. This case report supports the feasibility of single-shot spinal anesthesia in parturients with class III obesity and emphasizes the importance of rapid recognition and management of potential complications.

## Introduction

The prevalence of obesity is increasing worldwide, including in parturient patient populations. The WHO defines a normal BMI as 18.5-24.9 kg/m², overweight as 25-29.9 kg/m², and obesity as 30 kg/m² or higher. Obesity is further divided into three classes: class I (30-34.9), class II (35-39.9), and class III (40 or above). According to the American College of Obstetricians and Gynecologists, obesity is the most common medical condition in women of reproductive age [1]. It is strongly associated with comorbidities such as obstructive sleep apnea, hypertension, and diabetes mellitus [2].

Parturients, especially those with class III obesity, undergoing elective cesarean section carry a high perioperative risk as a result of obesity [2]. General anesthesia, while occasionally necessary, carries the risk of an anticipated difficult airway, aspiration, and postoperative respiratory complications. Neuraxial anesthesia offers advantages in minimizing these risks; however, it can be complicated by technical difficulties due to body habitus.

Here, we present a case report of the successful anesthetic management of cesarean section in a class III obese patient with single-shot spinal anesthesia, highlighting considerations and approaches for optimizing outcomes in this high-risk population.

## Case presentation

This is a 30-year-old woman who presented to our hospital for an elective cesarean section. She was 1.72 meters tall and weighed 180 kg, resulting in a BMI of 60.8 kg/m².

She had a previous preterm cesarean section seven years ago at 34 weeks due to preeclampsia. Her current pregnancy was complicated by gestational hypertension at 36 weeks. She was scheduled for an elective cesarean section one week after her diagnosis of gestational hypertension because she declined a trial of labor after cesarean delivery. Obstetric USG at 34 weeks confirmed vertex presentation of the singleton fetus with normal anatomy. Vital signs on admission were as follows: blood pressure 122/72 mmHg, heart rate 104 beats per minute, respiratory rate 19 breaths per minute, and body temperature 36.6 degrees Celsius. Laboratory tests, including complete blood count, comprehensive metabolic panel, and urinalysis, were within normal limits. Electrocardiography showed a normal sinus rhythm.

On pre-anesthesia evaluation, the patient was deemed American Society of Anesthesiologists (ASA) Physical Status Class 3 due to her elevated BMI. The airway examination revealed a Mallampati score of 1, which was reassuring given the wide mouth opening, normal neck extension, and a class I upper lip bite test. The spinous processes and interspaces could not be palpated. Spinal anesthesia was planned, and consent was obtained.

**Figure 1:**
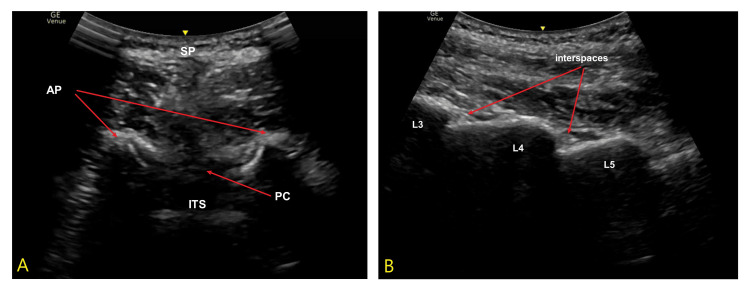
Pre-procedural neuraxial ultrasound examinations. Picture A (left) shows the transverse interlaminar view at the L3-L4 level. This view helps to identify the midline, confirm the interspaces, determine the needle entry angle, and estimate the depth of the intrathecal space. Picture B (right) shows the paramedian sagittal laminar view. The laminae of L3, L4, and L5 appear as a series of “steps,” with the interspaces between laminae visible. This view helps to identify the levels and interspaces. SP: Partial acoustic shadow from the spinous process; AP: Articular processes; PC: Posterior complex (ligamentum flavum + dura mater); ITS: Intrathecal space.

In the waiting area, we performed a pre-procedural neuraxial ultrasound exam to identify and mark the interspinous spaces in the sitting position, as shown in Figure 1.

**Figure 2:**
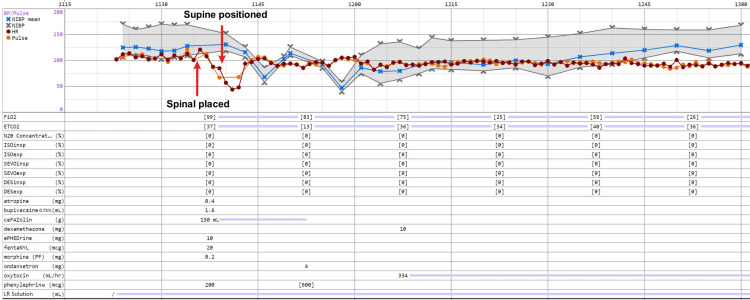
Intraoperative vital signs, relevant events, and medication administration timeline. The X-axis (time) is displayed in 15-minute increments. The Y-axis indicates blood pressure in mmHg and heart rate in beats per minute (bpm). The gray shaded area represents systolic/diastolic blood pressure ranges; blue crosses (×) and lines show mean NIBP, and red circles (●) indicate heart rate or pulse. Red arrows mark key events: spinal anesthesia placement and supine positioning. Medication doses (listed below) are shown in mg or mL at the time administered. ISOinsp, ISOexp, SEVOinsp, SEVOexp, DESinsp, and DESexp stand for inhaled and exhaled concentrations of isoflurane, sevoflurane, and desflurane. None of these general anesthetics were used; they are displayed as part of the anesthesia record by default in the electronic medical record system. BP: Blood pressure; NIBP: Noninvasive blood pressure; HR: Heart rate; FiO₂: Fraction of inspired oxygen; ETCO₂: End-tidal carbon dioxide.

The patient was then taken to the operating room after two units of packed RBCs were prepared. ASA standard monitoring was applied. The first set of vital signs recorded was as follows: blood pressure 170/107 mmHg (from cuff), normal sinus rhythm at a rate of 103 beats per minute, respiratory rate 19 breaths per minute, and oxygen saturation of 98% on room air. The patient was placed in a sitting position for spinal anesthesia. Spinal anesthesia was performed using a 22-gauge Pencan spinal needle at the L3-L4 interspinous space determined by the pre-procedure neuraxial ultrasound. CSF was observed after dural puncture. Twelve milligrams of 0.75% hyperbaric bupivacaine, 20 μg fentanyl, and 0.2 mg morphine were injected into the CSF on the first attempt. The patient was then placed in a supine position. Immediately after being placed supine, she reported nausea and vomited once. Her head was turned to the left, and all vomitus was suctioned. However, she became minimally responsive to verbal commands, and help was called. Her blood pressure after the spinal injection was 152/119 mmHg, which then decreased to 86/58 mmHg; her heart rate dropped from 88 to 44 beats per minute. She recovered within seconds after intravenous administration of 0.4 mg atropine, 10 mg ephedrine, and 200 μg phenylephrine. She became responsive and was able to follow commands. Her hand grip was firm, and her sensory blockade level was determined to be at T4 using an alcohol pad. Ten minutes after skin incision, a boy weighing 3920 g was delivered. The baby had Apgar scores of 3 and 7 at 1 and 5 minutes, respectively, and was assessed and resuscitated by the pediatrics team. The operation was uneventful, lasting about 75 minutes, with an estimated blood loss of 860 mL. A total of 1550 mL of lactated Ringer's solution was administered during the procedure. The patient did not report any pain during surgery, and no additional pain medications were required. She did not require further vasopressors after skin incision. The mother was transferred to the recovery room after stable vital signs were confirmed. She was discharged on postoperative day 1. She was later placed under observation on day 4 for chronic hypertension and discharged on day 5 after achieving good blood pressure control with medication. Detailed intraoperative vital signs, events, and medications used in this case are shown in Figure 2.

## Discussion

Class III obesity, formerly known as morbid obesity, is defined as a BMI of 40 kg/m² and above by the WHO, as mentioned above. Class III obese parturients are often associated with obstructive sleep apnea and obesity hypoventilation syndrome [2]. In addition, respiratory disorders, as well as other comorbidities such as diabetes mellitus, arterial hypertension, atrial fibrillation, and coronary artery disease, increase the risk of perioperative cardiopulmonary complications and therefore present additional challenges for anesthesia providers [2]. The choice of anesthesia for morbidly and super-morbidly obese parturients is debated. In obese patients, general anesthesia carries a significantly higher risk of difficult intubation, challenging ventilation, drug-induced respiratory depression, and cardiac complications [2].

Regional anesthesia for cesarean section includes single-shot spinal anesthesia, epidural anesthesia, and combined spinal-epidural anesthesia. Compared with general anesthesia, regional anesthesia offers several advantages, including reduced risk of airway and respiratory complications, faster recovery, improved postoperative pain control, reduced opioid use for postoperative analgesia, and less postoperative nausea and vomiting [2]. In addition, epidural anesthesia and combined spinal-epidural anesthesia can maintain adequate nerve block even during longer operating times. However, neuraxial anesthesia can be challenging in obese patients due to difficulty in palpation and identification of the epidural needle insertion space, uncertainty about the optimal dose, and increased risk of hemodynamic instability.

The use of ultrasound has emerged as a potential solution. Ultrasound helps visualize the spinous processes, determine the correct interspinous spaces, and measure the depth from the skin to the intrathecal space [3]. It can also be used in real time for needle insertion guidance. Some randomized controlled trials support its use, demonstrating increased first-attempt success rates, reduced needle redirections, and shorter procedure times [4,5]. However, other studies have not found significant differences compared to traditional palpation techniques [6,7], possibly due to variations in operator skill, patient body habitus, or study power. Moreover, ultrasound's utility is limited by reduced needle visibility, especially in class III obese patients, and it does not fully mitigate risks such as intravascular catheterization or dural puncture if epidural anesthesia is used [8,9]. In our case, we performed a pre-procedural ultrasound examination to identify the interspinous spaces, which later helped with the spinal anesthesia. With the help of the pre-determined spaces, we managed to achieve spinal anesthesia on the first attempt.

In terms of intrathecal dosage of local anesthetics, the optimal dose for class III obese patients remains unclear. With higher intrathecal doses, the success rate of blockade increases, but so does the risk of high spinal anesthesia or total spinal anesthesia. On the other hand, to achieve more stable hemodynamics, a reduced dose may be used by some anesthesiologists, but it is associated with a higher failure rate of blockade. Several studies have shown that the ED95 dose of local anesthetics in class III obese parturients is not affected by obesity. Lee Y et al., who studied patients with a mean BMI > 40 kg/m² and others with a mean BMI < 30 kg/m², suggested that the estimated ED95 doses in both groups were similar, at least 11.49 mg [10]. Another study by Carvalho B et al. supported these results. They found that the ED95 dose of bupivacaine, when administered with intrathecal fentanyl (10 µg) and morphine (200 µg), in morbidly obese parturients was 15 mg, which was similar to corresponding values in patients without obesity [11].

However, in obese patients with even higher BMIs, the evidence is lacking. A recent study that enrolled only parturients with BMI > 50 kg/m² demonstrated that the estimated ED90 of hyperbaric bupivacaine with fentanyl and morphine was approximately 11.5 mg [12], similar to the ED95 dose in non-obese and morbidly obese parturients. Some opinions suggest a reduced dose for super-obese patients to minimize symptoms of high spinal block, such as hypotension, nausea, and respiratory compromise. However, in Hon's study, all participants who experienced excessive cephalad block were asymptomatic and did not require conversion to general anesthesia [12]. Furthermore, although a reduced dose may initially achieve satisfactory blockade, morbidly or super-obese parturients are more likely to undergo prolonged surgeries, and a reduced dose is not recommended due to the increased risk of intraoperative anesthetic failure. A multicenter prospective cohort study involving parturients with morbid obesity found that patients undergoing cesarean section had a higher risk of failed spinal anesthesia (including the need for supplemental analgesia to complete surgery) when the bupivacaine dose was less than 10 mg [13]. Although a few case reports have documented successful management with reduced spinal doses, we believe there is selection bias, and the dose should not be reduced in obese patients with BMI > 50, as hemodynamic changes can be managed with vasopressor support.

Intrathecal morphine for cesarean section is considered superior for postoperative analgesia. It may raise concerns regarding postoperative respiratory depression, especially in patients at risk of hypoventilation. In a large retrospective study that included 5,036 patients with a mean BMI of 34 who received intrathecal morphine during cesarean section, there was no reported incidence of respiratory depression [14]. Based on the above studies, we used a standard dose of hyperbaric bupivacaine and intrathecal morphine for spinal anesthesia in this case.

Higher intra-abdominal pressure combined with a greater degree of inferior vena cava compression increases the risk of hypotension in the supine position. Significant hemodynamic fluctuations typically occur after patients lie in the supine position, as seen in our case. Elsakka AI et al. reported that remaining seated for two minutes after injection may reduce the incidence of hypotension compared to immediate supine positioning [15]. When patients are in the supine position, the left-lateral tilt is generally considered a useful method to alleviate hemodynamic effects.

Vasoactive drugs are also considered a preventive measure. Liu S et al. found that the left-lateral tilt position combined with prophylactic phenylephrine use is much more effective than maternal positioning alone [16]. When prophylactic phenylephrine infusion is administered after spinal injection to prevent hypotension, the appropriate dose should be adjusted based on maternal BMI. A prospective cohort study found that the ED50 of phenylephrine per kilogram in obese parturients was 0.49 μg/kg/min, significantly higher than in healthy patients [17]. However, the mean BMI of the obese group in that study was 32.8 kg/m², indicating that the result is not specific to obese parturients with higher BMIs. Future studies should focus on patients with higher BMI and aim to calculate ED90 or ED95 for more accurate clinical dosing. In our case, the patient developed hypotension seconds after being placed in the supine position, and her blood pressure improved quickly after vasopressor boluses. A better approach would have been to initiate a continuous vasopressor infusion and implement gradual positioning after spinal anesthesia.

This case reports the successful use of single-shot spinal anesthesia in a class III obese parturient with a BMI over 60 undergoing cesarean section, while also identifying areas for quality improvement. In our case, we utilized pre-procedural ultrasound to improve visualization, which facilitated successful spinal block placement on the first attempt. A standard dose of hyperbaric bupivacaine with intrathecal opioids provided effective anesthesia without the development of high spinal sensory blockade. However, dramatic hemodynamic changes occurred quickly after spinal injection and repositioning, which could have been mitigated by more gradual positioning and prophylactic vasopressor infusion. Combined spinal-epidural anesthesia is a reasonable alternative, as it allows a reduced initial spinal dose and the ability to extend and titrate through the epidural catheter during surgery.

## Conclusions

This case report emphasizes the use of ultrasound guidance for first-attempt success of neuraxial procedures, the importance of close monitoring for rapid hemodynamic changes following spinal anesthesia and repositioning, and the prophylactic use of vasopressor infusion to mitigate these dramatic changes. Further prospective studies are needed in obese parturients, especially those with a BMI greater than 50 kg/m², to better define optimal spinal dosing and protocols for preventing complications and improving patient outcomes.
